# PSGL-1, a Strategic Biomarker for Pathological Conditions in HIV Infection: A Hypothesis Review

**DOI:** 10.3390/v15112197

**Published:** 2023-10-31

**Authors:** Silvere D. Zaongo, Yaokai Chen

**Affiliations:** Department of Infectious Diseases, Chongqing Public Health Medical Center, Chongqing 400036, China; zsildieu@yahoo.fr

**Keywords:** HIV, P-selectin glycoprotein ligand-1 (PSGL-1), biomarker, HIV disease progression, cancer

## Abstract

P-selectin glycoprotein ligand-1 (PSGL-1) has been established to be a cell adhesion molecule that is involved in the cellular rolling mechanism and the extravasation cascade, enabling the recruitment of immune cells to sites of inflammation. In recent years, researchers have established that PSGL-1 also functions as an HIV restriction factor. PSGL-1 has been shown to inhibit the HIV reverse transcription process and inhibit the infectivity of HIV virions produced by cells expressing PSGL-1. Cumulative evidence gleaned from contemporary literature suggests that PSGL-1 expression negatively affects the functions of immune cells, particularly T-cells, which are critical participants in the defense against HIV infection. Indeed, some researchers have observed that PSGL-1 expression and signaling provokes T-cell exhaustion. Additionally, it has been established that PSGL-1 may also mediate virus capture and subsequent transfer to permissive cells. We therefore believe that, in addition to its beneficial roles, such as its function as a proinflammatory molecule and an HIV restriction factor, PSGL-1 expression during HIV infection may be disadvantageous and may potentially predict HIV disease progression. In this hypothesis review, we provide substantial discussions with respect to the possibility of using PSGL-1 to predict the potential development of particular pathological conditions commonly seen during HIV infection. Specifically, we speculate that PSGL-1 may possibly be a reliable biomarker for immunological status, inflammation/translocation, cell exhaustion, and the development of HIV-related cancers. Future investigations directed towards our hypotheses may help to evolve innovative strategies for the monitoring and/or treatment of HIV-infected individuals.

## 1. Introduction

In humans, P-selectin glycoprotein ligand-1 (PSGL-1) (or cluster differentiation (CD) 162) is coded by selectin P ligand (SELPLG). This cellular transmembrane receptor is expressed on most hematopoietic cells [1,2], including T-cells, B-cells, neutrophils, monocytes, and platelets [3]. PSGL-1 is a critical proinflammatory protein that is essential for the recruitment of immune cells into sites of inflammation. As such, PSGL-1 significantly contributes to the relocation of immune cells from the circulating blood to inflamed tissue. This process is well described and is referred to as extravasation. Indeed, past in vitro investigations have demonstrated that PSGL-1 mediates the relocation of neutrophils [4], CD8+ T-cells [5], CD4+ T-cells [6], macrophages/monocytes, plasma B-cells, and dendritic cells [7,8] into inflamed tissues through P-selectin (expressed on platelets) binding. The effects of PSGL-1 expression in infectious disease processes are also well documented [9]. For example, Kum et al. [10] reported that a robust response to *Salmonella typhimurium* infection requires PSGL-1/P-selectin interaction, which subsequently results in neutrophil recruitment. The beneficial roles of PSGL-1 in microbial infections by organisms such as SARS-CoV and SARS-CoV-2 [11], murine leukemia virus [12], and influenza virus [12] have also been reported. However, the precise roles of PSGL-1 in a chronic infection, such as HIV-1 infection, have not been described in the literature as yet and remain to be elucidated.

Several recent studies have demonstrated that PSGL-1 functions as an HIV restriction factor [13,14,15]. Researchers observed that PSGL-1 inhibits HIV-1 DNA synthesis and virion infectivity. In order to inhibit HIV-1 DNA synthesis, PSGL-1 interacts with actin (near the cell membrane), which is essential for the reverse transcription process [13]. Indeed, Liu et al. [15] demonstrated that PSGL-1′s highly conserved cytoplasmic residue (identified as threonine 393 (T393)) binds filamentous actin (F-actin) present near the cell membrane, blocks its depolymerization, and prevents actin recruitment by the HIV-1 gag MA domain to achieve cDNA synthesis. The role of T393 is critical, as a T393 mutation leads to a highly efficient reverse transcription process by HIV-1 [15]. Additionally, PSGL-1 is responsible for the release of membrane defective HIV-1 particles. As such, compared to an HIV-1-infected cell without PSGL-1, an infected cell in which PSGL-1 is expressed releases novel viral particles within which PSGL-1 is incorporated instead of crucial elements such as gp120 and gp41. Cognizant of the significant roles played by both gp120 and gp41 in the HIV infection process (cell attachment and membrane fusion, respectively), it is accurate to say that PSGL-1 not only represses HIV-1 replication but also reduces the infectivity of newly produced HIV-1 particles. However, the inhibition of virion infectivity by PSGL-1 requires the presence of its extracellular domain, as reported by Fu et al. [12]. Thus, the inhibition of HIV-1 DNA synthesis requires the conserved cytoplasmic domain (T393), whereas the inhibition of virion infectivity requires the extracellular domain. As observed by Liu et al. [14,15], and compared to HIV DNA synthesis, infectivity inhibition appears to be a more potent option utilized by PSGL-1 to antagonize the HIV-1 replication process. Furthermore, the preceding authors indicated that a relatively small degree of PSGL-1 overexpression may lead to a significant decline in virion infectivity [14].

PSGL-1 expression may also have additional, potentially disadvantageous ramifications during HIV infection. Indeed, Burnie et al. [16] found that, besides being an HIV restriction factor, PSGL-1 may mediate virus capture and subsequent transfer to permissive cells. Thus, the PSGL-1 that is incorporated into new virions may interact with P-selectin on uninfected cells and contribute to HIV entry within those cells. In parallel, past in vitro experiments by Levesque et al. [17] demonstrated that PSGL-1 expression and engagement with P-selectin or anti-PSGL-1 antibody may inhibit the proliferation of human hematopoietic stem cells (HSCs). This observation, combined with additional evidence that was reported in a review article by Tinocco et al. [2], led these researchers to postulate (in 2017) that PSGL-1 may modulate T-cell receptor (TCR) signals as an immune checkpoint inhibitor of T-cells. In 2023, Hope et al. [18] observed, on the one hand, that PSGL-1 attenuates TCR signaling and suppresses CD8+ T-cell progenitor differentiation. On the other hand, however, they also demonstrated that PSGL-1 provokes CD8+ T-cell exhaustion [18]. In light of the evidence reported by Tinocco et al. [2] and Hope et al. [18], and cognizant of the crucial roles played by T-cells in the immune response to HIV infection, PSGL-1 expression on these immune cells may therefore negatively modulate immune system responses. Thus, investigation of the additional roles that PSGL-1 plays during HIV infection is of particular interest. However, evidence suggests that PSGL-1 expression and signaling may also depend on other factors, such as the period of exposure/infection, ART treatment status, presence of co-infections, and other as yet unidentified factors. As indicated by several authors [19,20,21,22,23,24], these factors may significantly modulate inflammation and PSGL-1 (known to be a proinflammatory adhesion molecule) expression in HIV-infected individuals. We therefore believe that PSGL-1′s role as an HIV restriction factor may be limited to specific categories of HIV-infected individuals; however, these categories are yet to be clearly and categorically identified.

Since the introduction of modern antiretroviral therapy (ART), most appropriately treated HIV-infected individuals tend to eventually achieve undetectable viral loads, which implies that the effect of HIV on PSGL-1 expression (i.e., repression, which is further elaborated in hypothesis 1) may be minimal. However, researchers [25] have observed that, compared to those of healthy patients, monocytes derived from ART-treated HIV-infected individuals exhibit higher levels of PSGL-1. Such differences, when adequately and comprehensively analyzed, may aid in the elucidation of potentially novel roles that may be played by PSGL-1 during HIV infection. In this hypothesis review, we propose to provide interpretations of PSGL-1 functional expression before and after ART administration. Our hypotheses offer speculation on the potential utilization of PSGL-1 as a marker for immune suppression, a marker for inflammation and/or translocation, a marker for cell exhaustion, and a marker for potential or latent neoplastic disease during HIV infection. If validated in future research, these hypotheses could potentially lead to the development of novel strategies for the monitoring and treatment of HIV-infected individuals.

## 2. Hypothesis 1: A Marker of Immune Depletion Prior to ART Initiation

**Figure 1:**
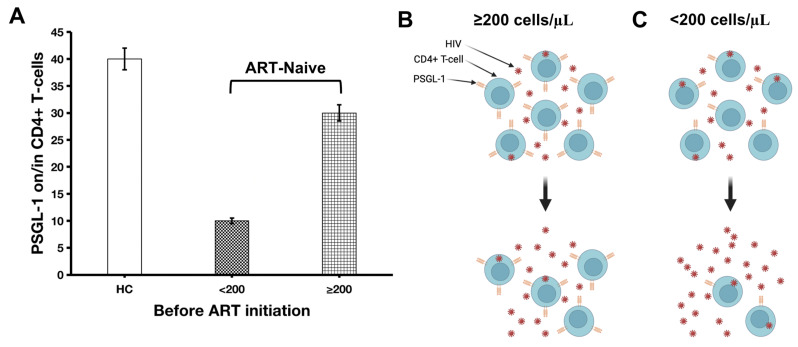
PSGL-1 expression before ART initiation (**A**) and the potential impact of PSGL-1 expression on CD4+ T-cell counts (**B**,**C**). In (**A**), we speculate that, compared to healthy controls, PSGL-1 expression is significantly repressed in ART-naïve individuals. However, HIV-positive individuals with very low CD4+ T-cell counts (<200 cells/µL) are likely to have significantly lower levels of PSGL-1 compared to those with CD4+ T-cell counts ≥200 cells/µL. (**B**) represents ART-naïve individuals who have CD4+ T-cell counts ≥200/µL. Within this stratum, PSGL-1 is repressed but remains at a level that is essential to responding to HIV infection. As PSGL-1 becomes fully repressed, more CD4+ T-cells become infected, as the virions in (**C**) are more competent at infecting target CD4+ T-cells than those in (**B**). Consequently, via the functioning of CD8+ T-cells, NK cells, and apoptosis, the infected CD4+ T-cells will be progressively eliminated. Ultimately, we are of the opinion that, prior to ART initiation, patients that have a higher degree of PSGL-1 expression on CD4+ T-cells will likely have higher CD4+ T-cell counts and lower HIV viral loads. Our hypothesis is further supported by a study by Burnie et al. [32] that demonstrated that virions from T-cells (and PBMCs in general) expressing low levels of PSGL-1 contain low levels of PSGL-1 and high levels of gp120 and gp41. These virions may mediate infection of new cells through P-selectin binding and lead to higher HIV viral loads. In other words, cells expressing lower levels of PSGL-1 in the context of HIV infection do not necessarily produce noninfective virions. However, virions from cells expressing high levels of PSGL-1 (i) display high levels of PSGL-1, (ii) have low levels of gp41 and gp120, and (iii) are incompetent at infecting new target cells even via P-selectin binding with new cells. HC: healthy control; <200: individuals with CD4+ T-cells < 200 cells/µL; ≥200: individuals with CD4+ T-cells ≥ 200 cells/µL.

Information gleaned from the contemporary literature indicates that PSGL-1 is an HIV restriction factor and HIV, in turn, utilizes several mechanisms to counter PSGL-1 activity. As such, it has been reported that, in order to readily replicate in infected cells and to infect new cells without restraint, HIV-1 induces a downregulation of PSGL-1 [25]. Nef negatively regulates extracellular PSGL-1 levels [12], whereas viral protein U (Vpu) negatively influences both intra- and extracellular levels of PSGL-1 [12,15]. Vpu, as observed by Liu et al., binds to PSGL-1 and induces its ubiquitination and degradation via the ubiquitin ligase SCF^β-TrCP2^ protein complex [15]. Therefore, when compared to healthy controls, PSGL-1 expression in ART-naïve HIV-infected individuals is likely to be significantly repressed (Figure 1A). This is supported by Liang et al. [25] who observed that HIV-infected individuals have significantly lower levels of PSGL-1 expressed on monocytes compared to healthy controls. However, even if PSGL-1 does not directly repress HIV replication, PSGL-1 incorporation into new virions may negatively influence HIV viral loads due to the reduced capacity for infection of new target cells.

Following the logic described above, HIV viral loads in ART-naïve HIV positive individuals may be associated with PSGL-1 expression. We believe that there is a negative correlation between levels of individual HIV virions and PSGL-1 levels on immune cells, particularly on T-cells. This is supported by observations reported by Liang et al. [25], who noted that the viral load profile (log10 copies/mL) in treatment-naïve participants with primary HIV-1 infection (PHI) was approximately four times higher than in a cohort of participants with chronic HIV infection (CHI) (4.21 ± 0.95 versus 1.30 ± 1.68, respectively). Notably, PSGL-1 levels on monocytes collected from the PHI cohort were significantly reduced compared to those in the CHI cohort. It is important to remember that PSGL-1, like other HIV restriction factors, conforms to important characteristics such as (i) induction by interferons [26], (ii) response to lentivirus infection by significant production of its amino acid sequences [27], and (iii) having its activities countered by lentivirus (in the case of HIV infection, Vpu and Nef are essential to countering PSGL-1 activities). Thus, the success of PSGL-1 anti-HIV activities prior to any ART commencement will likely be translated into a more robust immune response, manifested by higher CD4+ T-cell counts and lower HIV viral loads. Indeed, if PSGL-1 produces virions not competent at infecting new target cells (preferentially CD4+ T-cells), it is therefore rational that patients with higher PSGL-1 levels will also have higher CD4+ T-cell counts (Figure 1A,B) and lower HIV RNA loads. This would be a consequence of the reduction in infectivity caused by the production of defective HIV virions. In the study by Liang et al. [25], CD4+ T-cell counts in the PHI cohort were relatively lower than those in the CHI cohort (432.56 ± 147.2 versus 474.06 ± 195.7, respectively). We believe that, had further stratification within each group (PHI and CHI) been carried out based on a period of exposure/infection, it may have provided clearer evidence regarding the differences in terms of CD4+ T-cells in those two groups. Unfortunately, studies and data related to PSGL-1 expression in ART-naïve individuals are limited. To our knowledge, the study by Liang et al. [25] is unique. Nonetheless, taking into consideration the preceding data, we are of the opinion that PSGL-1 may well be seen as a biomarker for immune depletion in HIV patients who are not receiving ART. Future investigations on PSGL-1 expression (polymerase chain reaction (PCR) for mRNA expression, and flow cytometry for PSGL-1 extracellular expression) in HIV-infected ART-naïve individuals will be necessary in order to validate our hypothesis.

Blood samples from ART-naïve individuals may be classified based on their CD4+ T-cell counts. For example, we may identify two groups, such as patients with CD4+ T-cell counts <200 cells/µL and those with CD4+ T-cell counts ≥200 cells/µL. We are of the opinion that patients with CD4+ T-cell counts <200 cells/µL will display significantly high HIV viral loads and lower PSGL-1 levels compared to those with ≥200 CD4+ T-cells/µL. To illustrate this, it is known that patients with CD4+ T-cell counts <200 cells/µL display weaker immune response to infections compared to their counterparts with CD4+ T-cells ≥ 200 cells/µL [28,29,30]. In this context, associated with the absence of ART, HIV viral loads are also known to be significantly increased in patients with CD4+ T-cells < 200 cells/µL, as shown by Fox et al. [31] (the relative risk of having a baseline viral load of ≥ 100,000 in patients with CD4+ T-cell counts <200 cells/µL was 1.67 (1.43–1.73)). These large quantities of circulating copies of HIV may contribute to significantly repressing PSGL-1 on CD4+ T-cells in patients with CD4+ T-cell counts <200 cells/µL. Interestingly, the findings by Burnie et al. [32] indicated that a productive HIV infection requires highly repressed PSGL-1 on infected T-cells or PBMCs, which in turn leads to the production of virions displaying low levels of PSGL-1 and high levels of gp120 and gp41. Indeed, this type of virion remains fully infectious, as they may even mediate HIV infection via PSGL-1/P-selectin binding. In the reverse scenario, when cells express high levels of PSGL-1, the virions they produce contain high levels of PSGL-1, low levels of gp120, and low levels of gp41. Virions from such cells are therefore not as competent at infecting new target cells. This may occur in the context of low HIV viral loads, with cells expressing significantly higher levels of PSGL-1. Thus, even before future conclusive evidence, it may be expected that ART-naïve patients with CD4+ T-cell counts of <200 cells/µL will likely display significantly higher HIV viral loads and lower PSGL-1 levels compared to those with CD4+ T-cell counts of ≥200 cells/µL. However, one particular selection bias needs to be controlled during this investigative process. If the period of infection is not determined and matched between the two groups, patients infected relatively recently will likely display higher CD4+ T-cell counts than those having been infected for a much longer period. In this scenario, the period of infection will distort the interpretation of PSGL-1 expression and jeopardize its utilization as a potential marker of immune suppression. Additionally, particular attention should be placed on the absence of any other co-existing chronic infective process. Indeed, since PSGL-1 is a proinflammatory protein, its expression during HIV infection may not be appropriately appreciated if another chronic disease is present in the selected study population. We believe, also, that more virulent strains of HIV-1 are capable of repressing PSGL-1 expression with higher efficiency than others. In China, for example, it has been observed that patients infected with the CRF_01 AE clade have inherently lower CD4+ T-cell counts [33,34], poor survival, and more rapid HIV disease progression than non-CRF_01 AE clades [34,35,36]. As such, we believe that Vpu derived from the CRF_01 AE clade may have a stronger binding affinity and a greater capability to repress PSGL-1 than the CRF_07 BC clade, and this may manifest as the relatively lower CD4+ T-cell counts observed in patients infected by this strain.

## 3. Hypothesis 2: A Marker of Immune Reconstitution after ART Initiation

In most ART-treated individuals, HIV viral loads eventually become undetectable (<50 copies/mL), and as such, we believe that the direct effect of HIV on PSGL-1 repression may be considered to be minimal. Moreover, we are of the opinion that, in this category of HIV-infected individuals, PSGL-1 expression may be sustained by the persistent inflammatory state resulting from HIV infection. Indeed, PSGL-1 overexpression has been intriguingly linked with inflammation, as observed during the studies of Almulki et al. [37], Somers et al. [38], and others [39,40]. HIV infection itself is also known for initiating and sustaining chronic inflammation [41], which emerges secondary to a gastrointestinal breach, also referred to as a leaky gut, which tends to occur early in HIV infection [42,43]. Interestingly, PSGL-1 overexpression has been demonstrated in HIV-infected adults receiving ART. Compared to PSGL-1 levels on monocytes from healthy donors, Connor et al. [24] observed higher values of PSGL-1 on monocytes from HIV-infected individuals. This profile (i) confirms the minimal effects that HIV Vpu and Nef play in PSGL-1 expression and (ii) suggests that the inherent chronic inflammation may be the primary driver of PSGL-1 expression in these categories of HIV-positive individuals. In the preceding scenario, we asked ourselves what the precise role of PSGL-1 may be in patients receiving ART. Does the overexpression of PSGL-1, a well-known proinflammatory protein, in ART-treated individuals have significant meaning? Our answer to this question is in the affirmative. Indeed, we are of the opinion that PSGL-1 levels may determine the immune recovery process in ART-treated individuals. The fundamental mechanisms underlying incomplete immune recovery are not fully understood; however, several inflammation-related proteins have been associated with T-cell immune recovery during HIV infection [44]. For example, CUB domain-containing protein 1 (CDCP1), C-X-C motif chemokine 11 (CXCL11), cystatin-D (CST5), signaling lymphocytic activation molecule 1 (SLAMF1), TNF-related activation-induced cytokine (TRANCE), and CD5 have all been suggested to be potentially useful for distinguishing immunological responders (IRs) from immunological non-responders (INRs) [45]. Specifically, Wan et al. [45] observed that (i) levels of CDCP1, CXCL11, CST5, and SLAMF1 were higher in INRs than in IRs and that (ii) levels of TRANCE and CD5 were lower in INRs compared to IRs. Thus, in the different contexts of inflammation seen between IRs and INRs (a higher inflammation profile has been reported in INRs [46,47,48,49,50,51,52]), PSGL-1 may be one more inflammatory protein potentially contributing to immune cell recovery or depletion in ART-treated HIV-positive individuals. Unfortunately, as yet, our assertion has no supporting data (or contradictory data, for that matter) in contemporarily published literature, and only future pioneering investigations are likely to contribute to the elucidation of the role of PSGL-1 in the immune recovery process.

**Figure 2:**
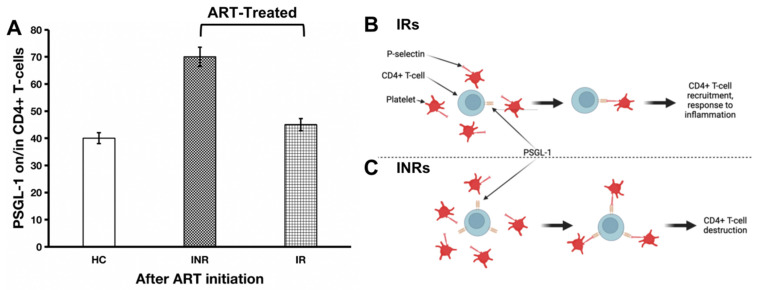
PSGL-1 expression after ART initiation (**A**) and its implications for CD4+ T-cell destruction in HIV-positive individuals receiving ART. In IRs (**B**), the lower rate of inflammation is manifested by low levels of PSGL-1 expression (which are comparable to levels of inflammation in healthy controls). In this context, PSGL-1 will help with recruiting CD4+ T-cells to the site of inflammation. In contrast, in INRs (**C**), despite ART administration, the level of inflammation remains significantly elevated. In this scenario, excess PSGL-1 will lead to engagement with platelets. Ultimately, these PSGL-1/platelet aggregates entrap CD4+ T-cells and lead to their destruction. This hypothesis will require exploration and validation by future studies. HC: healthy control; INR: immunological nonresponder; IR: immunological responder.

Our literature review informs us that, among ART-treated individuals, 10 to 40% of patients do not achieve complete immune reconstitution and are referred to as immunological nonresponders (INRs). An adequate immunological response to ART can be defined as an increase in CD4+ T-cell counts to > 500 cells/µL, owing to the fact that morbidity and mortality rates in PLWH with CD4+ T-cell counts > 500 CD4+ T-cells/µL are equivalent to those rates in HIV-negative individuals [53,54,55,56]. There is no standardized definition of INRs or IRs; however, it may be assumed that patients treated with ART for more than two years who achieve undetectable HIV viral loads and who achieve a CD4+ T-cell count of less than 200 cells/µL may be referred to as INRs [57]. In other words, those with CD4+ T-cell counts ≥ 200 cells/µL may be defined as IRs. Interestingly, researchers have observed that markers of inflammation such as sCD163 and sCD14 are highly expressed in INRs compared to IRs [58]. It is plausible that PSGL-1 expression in INRs follows a similar trend. As such, we believe that, compared to INRs, PSGL-1 expression in IRs is significantly lower (Figure 2A). The expected lower levels of PSGL-1 in IRs compared to INRs may also be regarded as an indicator of lower levels of inflammation in IRs. The ultimate consequence of such an overexpression of PSGL-1 in INRs, in our opinion, is to promote the formation of CD4+ T-cells and platelet aggregates through PSGL-1/P-selectin binding (Figure 2B,C). As observed by Dai et al. [58], compared to IRs, a greater degree of CD4+ T-cell/platelet aggregates is found in INRs, and the formation of CD4+ T-cell/platelet and/or CD4+ T-cell/endothelial cell aggregates may negatively influence CD4+ T-cell survival. Indeed, Dai et al. [58] demonstrated that increased levels of CD4+ T-cell/platelet aggregates correlate with HIV-1 permissiveness and CD4+ T-cell loss. Notably, Zarbock et al. [59] demonstrated that PSGL-1 may promote the death of activated T-cells via a caspase-independent pathway involving apoptotic mediators, such as apoptosis-inducing factor (AIF) and cytochrome c complex [60]. Interestingly, Dai et al. [58] observed high levels of caspase-1 and caspase-3 and low levels of Bcl-2 in CD4+ T-cell/platelet aggregates isolated from INRs. These reports suggest a potential negative role that may be played by PSGL-1 in CD4+ T-cell destruction and in the incomplete immune reconstitution process. Further investigations will be necessary to confirm whether PSGL-1 may be considered a reliable marker of immune reconstitution in ART-treated individuals.

## 4. Hypothesis 3: A Marker of Inflammation and/or Translocation

**Figure 3:**
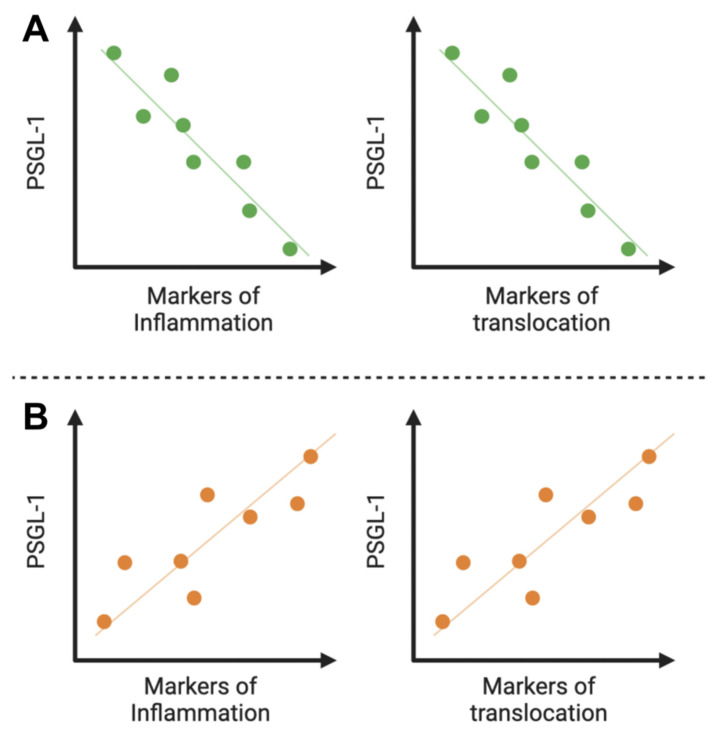
Potential correlation between PSGL-1 and markers of inflammation and/or microbial translocation. Prior to ART initiation (**A**), we believe that there is a negative association between PSGL-1 and important biomarkers such as sCD163, sCD14, sCD40, LPS, and β-glucan. However, ART initiation, which leads to low/undetectable viral loads, may likely invert the association between PSGL-1 and markers of inflammation and/or microbial translocation (**B**). Thus, as plasma markers of inflammation and/or microbial translocation increase, PSGL-1 levels correspondingly increase. Thus, by implication, when levels decrease, PSGL-1 expression is also expected to decline.

As mentioned above (Figure 1A), prior to ART initiation, we believe that higher levels of PSGL-1 will be seen in patients with CD4+ T-cells ≥ 200 cells/µL. From this hypothesis, we speculate that this may indicate that levels of inflammation in these patients are lower than in patients with CD4+ T-cells < 200 cells/µL who display lower levels of PSGL-1 on their immune cells (CD4+ T-cells, in particular). However, as PSGL-1 is a proinflammatory protein [61], it remains challenging to understand why higher expression of PSGL-1 may reflect lower levels of inflammation. We believe this is a particular effect of HIV infection. Indeed, in patients with higher levels of CD4+ T-cells, the immune system is found to continue to struggle to control inflammation, and higher expression of PSGL-1 participates in this process. However, over time, the immune system becomes exhausted, and the persistent inflammation, combined with very high viral loads working on the downregulation of PSGL-1 expression, may explain why, prior to ART initiation, lower PSGL-1 levels may account for elevated serum inflammatory profiles (Figure 3A). In order to confirm this hypothesis, we believe that further investigations into plasma markers of inflammation such as soluble CD14 (sCD14), sCD163, and sCD40 should be conducted. Since the chronic inflammation seen during HIV infection has been shown to be a process primarily driven by microbial translocated products [62], we believe that, prior to ART initiation, lower PSGL-1 levels will also correlate with high levels of serum microbial translocation profiles (Figure 3A). Thus, studies into plasma markers of microbial translocation, such as beta (β)-glucan and lipopolysaccharides (LPS), are also necessary. We speculate that, compared to individuals with CD4+ T-cell counts of ≥200 cells/µL, the preceding markers (of inflammation and translocation) will likely be elevated in patients with CD4+ T-cells < 200 cells/µL. In the present era of widely used modern ART to treat HIV infection and also because of ethical considerations, it remains challenging to unearth data that present clear evidence of the relationships between CD4+ T-cell counts, the progression of plasma marker profiles of inflammation and translocation, and HIV disease progression over time. Despite the dearth of evidence supporting our preceding assertion, it is important to note that the progression of HIV infection is inextricably entwined with changes to CD4+ T-cell counts [63]. Indeed, CD4+ T-cells are the pivotal mediators for both cellular and humoral immune responses [64,65]. Unfortunately, infection of CD4+ T-cells by HIV results in their destruction (via apoptosis [66], pyroptosis [67], and/or cytotoxic T-cells [68,69]), which further augments the absence of a robust immunological response against HIV infection. Concomitantly, circulating HIV provokes an increasing and sustained production of proinflammatory cytokines and proteins resulting from the activation of immune cells [70]. Also, evidence has shown that the leaky gut syndrome that begins early in HIV infection [42,43] further advances as HIV disease progresses and promotes the further translocation of microbial products into the bloodstream [71]. In light of the preceding information, we believe that lower CD4+ T-cell counts may well be seen to be associated with higher levels of plasma biomarkers of inflammation and microbial translocation. Furthermore, if we can show, in future studies, that prior to ART commencement there is a negative correlation between the levels of these markers of inflammation/translocation and PSGL-1 expression at different strata of CD4+ T-cell counts, then the role of PSGL-1 as a potential marker of inflammation and/or translocation during HIV infection may acquire important research value.

In ART-treated individuals, we also believe that PSGL-1 may be used as a marker of inflammation and/or translocation. In Figure 2A, we predict that PSGL-1 levels in INRs are significantly higher than those observed in IRs. Indeed, we are of the opinion that inflammation and/or translocation markers have significantly higher values in INRs compared to IRs. This is supported by the observations of several research teams who have shown that markers of inflammation and/or microbial translocation (sCD40, sCD163, sCD14, LPS, β-glucan) have higher values in INRs compared to IRs [58,72,73,74]. Thus, it may well be valid to state that high levels of PSGL-1 in INRs are sustained by high levels of inflammation and/or microbial translocation. Therefore, the likelihood of observing a positive correlation between PSGL-1 expression and hematological biomarkers of inflammation and/or translocation in ART-treated individuals (both INRs and IRs) may well be high (Figure 3B). In other words, low values of PSGL-1 may correlate with low levels of plasma markers (inflammation and/or translocation) in IRs, and high values of PSGL-1 may correlate with high levels of plasma markers (of inflammation and/or translocation) in INRs. However, this hypothesis will require research validation by unambiguous evidence gleaned from future clinical investigations.

## 5. Hypothesis 4: A Marker of Cell Exhaustion in ART-Treated Individuals

One of the major characteristics of HIV infection, other than the chronic systemic inflammation [75] and the creation and persistence of reservoir cells [76,77], is the presence of transcriptomic and proteomic exhaustion signatures in and on T-cells (receptor or co-receptor) [78]. As defined by Fenwick et al. [78], T-cell exhaustion is a condition causing a progressive loss in T-cell effector function, during which an increased level of expression and an assortment of immune checkpoint inhibitors is seen to become evident. This is responsible for diminishing T-cell effector function, rendering these cells less potent in their response to HIV infection. For example, Wang et al. observed that exhausted CD8+ T-cells present fewer effector function phenotypes than CD8+ T-cells in HIV-negative individuals [79]. Within these exhausted cells, researchers have identified specific genes, such as killer cell lectin-like receptor subfamily G member 1 (KLRG1), cluster differentiation (CD160), T-cell immunoreceptor with Ig and ITIM domains (TIGIT), lymphocyte-activation gene 3 (LAG3), programmed death 1 (PD1), and CTL-4, that are associated with T-cell exhaustion [79,80,81]. Therefore, exhausted immune cells such as CD4+ T-cells, CD8+ T-cells, and natural killer cells (NK cells), among others, may alter their inherent functions and initiate aberrant behavior while constantly expressing exhaustion signatures. We are of the opinion that, during HIV infection, PSGL-1 (which has also been observed to be an immune checkpoint inhibitor for T-cells [82,83]) may be a reliable potential biomarker of cellular exhaustion during HIV infection (particularly in ART-treated individuals). Thus, this specific potential role of PSGL-1 should be further considered in future investigations.

It has been demonstrated that PSGL-1 signaling (PSGL-1 binding to P-selectin or E-selectin, or antibody crosslinking of PSGL-1) may promote effector T-cell exhaustion during chronic viral infections [83]. Tinocco et al. [83], on the one hand, demonstrated that a genetic deletion of PSGL-1 prevents the development of exhausted T-cells. On the other hand, CD8+ T-cells with deleted PSGL-1 express their effector function, which is also associated with viral clearance in the chronic lymphocytic choriomeningitis virus (LCVM) clone 13 model [83]. In the context of LCVM, it has been shown that PSGL-1 expression, even in the absence of selectin binding, is a significant contributor to T-cell exhaustion [83]. In another study, Tinocco et al. [84] observed that PSGL-1 deficiency secondary to acute LCVM Armstrong infection promotes greater CD8+ effector T-cell functions and memory progenitor T-cell production. Hope et al. [17] recently observed that PSGL-1, in addition to attenuation of TCR signaling, may also suppress CD8+ T-cell progenitor differentiation. Furthermore, the preceding investigators also demonstrated that PSGL-1 provokes CD8+ T-cell exhaustion [17]. These studies illustrate the fundamental role that PSGL-1 plays as a potential regulator of T-cell exhaustion and, above all, as a regulator of T-cell responses. In light of the critical roles played by both CD4+ and CD8+ T-cells during HIV infection, enhanced PSGL-1 expression is likely to provoke the exhaustion and loss of function of these cells, which may result in the vigorous progression of HIV infection.

**Figure 4:**
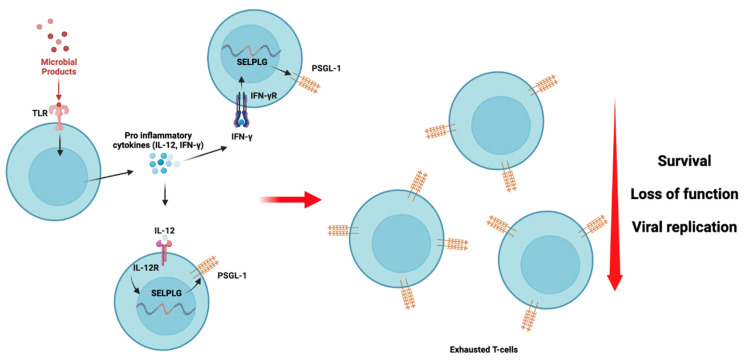
PSGL-1 signaling and its repercussions on T-cell exhaustion are potentially mediated by translocated microbial products and proinflammatory cytokine stimuli. Toll-like receptor (TLR, likely TLR4) responds to microbial stimuli by inducing the production of pro-inflammatory cytokines [86,87] such as IL-12 and IFN-γ. These cytokines may interact with their specific receptors and induce the production and expression of PSGL-1, as has been demonstrated previously [15,82]. As the translocated microbial product stimulus is sustained and persistent despite ART administration, we believe that T-cells in HIV-infected individuals express PSGL-1 continuously as long as the inflammation is sustained. Consequently, T-cells with PSGL-1 exhaustion signatures undergo loss of function and reduced survival, which results in their inability to effectively limit viral replication, as was observed by Tinocco et al. [83].

It has been established in ART-treated HIV-infected individuals that translocated microbial products continuously circulate in the bloodstream and provoke and sustain chronic inflammation [72]. During HIV infection, T-cell exhaustion can be seen as a consequence of chronic and sustained antigenic stimulation [85]. However, in patients with undetectable HIV viral loads, we believe that translocated microbial products may also induce a sustained expression of PSGL-1 (Figure 4), which may promote T-cell exhaustion. Thus far, we have found no published data in the literature presenting or discussing this hypothesis. However, it is known that interferon gamma (IFN-γ) [15], sCD40 ligand (sCD40L) [24], interleukin (IL)-12 [82], and glutamate [24] may trigger PSGL-1 expression on immune cells. In a recent publication by Hope et al. [17], it was observed that PSGL-1 blockade may reinvigorate exhausted T-cells. Thus, PSGL-1 represents a potential new target to suppress T-cell exhaustion in HIV-infected patients, particularly in those non-responsive to CTL-4 and/or PD-1 immune checkpoint blockade. Further investigations are warranted in this regard, particularly in the context of HIV infection, to validate this hypothesis.

## 6. Hypothesis 5: A Marker of Potential or Latent Cancer in INRs

Patients who do not achieve complete immune recovery after modern ART, also referred to as immunological nonresponders (CD4+ T-cell/µL < 500 cells/µL [88,89], <350 cells/µL [90,91], <200cells/µL [92], <100 cells/µL [93], or <50 cells/µL [94]), are more likely to succumb to non-AIDS comorbidities [53,95]. Among the non-AIDS comorbidities, there are several neoplastic diseases (liver cancer, anal cancer, cervical cancer, non-Hodgkin lymphoma) that preferentially afflict INRs [96,97,98]. We are of the opinion that both chronic inflammation associated with microbial translocation and the sustained expression of PSGL-1 leading to immune cell exhaustion represent a tandem that may potentially be responsible for the higher rates of non-AIDS comorbidities, and particularly cancers, in HIV-infected individuals. In the recent past, Lin et al. referred to PSGL-1 as a novel tumor microenvironment prognostic biomarker [99] and a potential immunotherapeutic target for cervical cancer. Indeed, they observed that cervical specimens with high-grade squamous lesions have overexpression of PSGL-1, and they also suggested that greater expression of PSGL-1 (mRNA ≥ 0.245) may be a promising predictor of cervical pre-malignancy and cancer [100]. Even though their samples were not harvested from HIV-positive individuals, the observations of Lin et al. [99] are illustrative of the potential role played by PSGL-1 during the onset of cancers. Similarly, several other studies point out significant associations between cancers and PSGL-1. Indeed, researchers have reported that interactions of selectins and PSGL-1 constitute a pivotal cause of cancer progression [100]. As such, on the one hand, it is acknowledged that cancer cells express selectin ligands (mucins, PSGL-1). On the other hand, the tumor itself rarely expresses selectin. This helps tumor cells to exploit interactions (selectin ligands/selectin binding) with normal blood cells or endothelial cells (which express selectins) to seed distant metastases. For instance, Dimitroff et al. [101] demonstrated that PSGL-1, in tandem with E-selectin ligand-1 (ESL-1), is involved in the development of prostate cancer bone metastases. They also observed that PSGL-1 is detectable on the surface of bone-metastatic prostate tumor cells. Moreover, the role of PSGL-1 in lung cancer was elucidated by Heidemann et al. [102]. Explicitly, Heidemann and her colleagues showed that selectin and selectin ligand (including PSGL-1) interactions are critical for small cell lung cancer cells to seed distant metastases. In their studies, they also observed that metastasis formation is not completely abrogated in selectin-deficient mice (E-/P-selectin-deficient mice), suggesting the complementary role played by selectin ligands that results in this outcome. Additionally, Hoss et al. [103] observed that, as cancer cells express high levels of PSGL-1, they may easily bind to selectins (P-selectin, E-selectin, L-selectin) on the surface of blood cells to (i) evade immune system suppression, (ii) promote extravasation to constantly evade immune system control, and (iii) promote metastasis. Moreover, it has been reported that PSGL-1 deficiency enables CD8+ T-cells to respond to low-affinity TCR ligand. This phenotype results, to an extent, in the inhibition of the growth of PD-1-blockade-resistant melanoma by enabling tumor-infiltrating T-cells [17]. Hope et al. [17] suggested that pharmacological blockade of PSGL-1 may represent an immunotherapeutic option for PD-1-blockade-resistant tumors, as this blockade helps to diminish T-cell exhaustion. We also believe that, in ART-treated INRs, immune cells with higher levels of PSGL-1 may be unable to play an anti-tumor role. Indeed, as a T-cell-intrinsic checkpoint regulator of exhaustion, PSGL-1 may mediate the malfunctioning of key anti-tumor cells. In this context, and particularly during HIV infection (which is known to induce permanent exhaustion of immune cells), a profiling of PSGL-1 signatures in specific organs may possibly assist with the identification and/or prevention of the onset of cancers.

## 7. Additional Challenges

In the first hypothesis proposed in this article, we mentioned that quantifying PSGL-1 levels by qPCR (in order to obtain mRNA levels) and flow cytometry (for the measurement of cell-bound PSGL-1) may be used as a marker of immune depletion in HIV-infected individuals. It is important to note that this initially refers to a profiling of PSGL-1 on/in CD4+ T-cells, which are the primary targets of HIV [104]. However, a broader investigative approach towards PSGL-1 levels on/in PBMCs in general may be necessary as well, should results be promising. As such, the utilization of tools such as single-cell sequencing may provide interesting results related to PSGL-1 expression on/in different varieties of immune cells (CD4+, CD8+, natural killers, monocytes, HIV+ reservoir cells, etc.) and, by extension, within different categories of HIV-infected individuals (acute-phase, chronic-phase, ART-treated, ART-naïve). Cell mapping based on PSGL-1 expression may aid in predicting the evolution of pathological conditions and ultimately preventing them. Above all, should PSGL-1 be established in the future as a critical marker during HIV infection, it is possible that the utilization of PBMCs for the profiling of PSGL-1 in low socio-economic settings may be easier and more practical, as PBMCs sampling is easier and isolation of CD4+ T-cells from PBMCs is not a procedure performed routinely in disadvantaged settings.

Screening for PSGL-1 to establish the potential relationships of PSGL-1 with common pathological conditions seen during HIV infection requires more robust understanding with respect to a few confounding factors. For example, the influence of body mass index (BMI) (particularly inflammation resulting from adipose tissue) on PSGL-1 expression may represent one major challenge to the utilization of PSGL-1 screening during HIV infection. Indeed, being overweight/obese induces an inflammatory state associated with the expression of diverse proinflammatory molecules within the body [105,106,107]. Whether PSGL-1 expression is also affected by elevated BMI is unknown. It has already been reported that PSGL-1 deficiency is protective against obesity-related insulin resistance [108]. The preceding study (performed on mice) revealed that, in a state of obesity, mice that express low levels of PSGL-1 are protected from developing obesity-related insulin resistance. Additionally, low levels of PSGL-1 have been shown to be associated with decreased macrophage infiltration and inflammation [108], suggesting that PSGL-1 levels are potentially upregulated in individuals displaying body mass indices that are within the overweight or obese range. Should BMI be found to influence PSGL-1 levels, it may be regarded as an important confounding factor, as it is also known that ART-treated individuals are inherently more likely to become obese/overweight. For example, in the United States of America, it has been observed that 22% and 5% of HIV-positive patients receiving ART became overweight and obese, respectively [109]. Therefore, further targeted study is warranted to provide a definitive answer to the preceding question.

The influence of age on PSGL-1 expression also requires further investigative work. Similar to BMI (overweight/obese), advancing age is also associated with a proinflammatory state [110]. Thus, it is legitimate to question the influence of age on PSGL-1 expression. Li et al. [110] reported that, during the aging process, immune cells such as CD4+ T-cells and CD8+ T-cells highly express IFNγ. However, IFN-γ has been shown to promote PSGL-1 expression, as demonstrated by Liu et al. [14]. It is therefore possible that immune cell samples collected from older persons may present higher levels of PSGL-1. This hypothesis, if proven to be valid, may help to confidently include age as another major confounding factor influencing PSGL-1 expression.

## 8. Conclusions

In conclusion, our hypotheses illustrate that, other than its established proinflammatory and HIV restriction factor functions, PSGL-1 may well be involved in other roles during HIV infection. Indeed, we believe that PSGL-1 exerts a beneficial effect prior to ART commencement, especially in the early stages of HIV infection. In the long term, however, and in the context of chronic inflammation, the consequences of PSGL-1 expression and signaling may become unfavorable. Evidence gleaned from the literature suggests the potential roles of PSGL-1 in immune depletion, immune reconstitution, inflammation/translocation, cell exhaustion, and cancers. Our preceding hypotheses are theoretical, and as such, further investigations will be necessary to evaluate whether PSGL-1 may be a reliable biomarker for some of the aforementioned pathological conditions common to HIV-infected individuals. The results of studies investigating the preceding hypotheses may potentially represent major breakthroughs and would establish the groundwork with respect to the future development of effective novel therapeutic strategies to successfully improve and restore CD4+ T-cell counts in INRs, to prevent HIV-related cancers, or to potentially cure certain cancers.

## Data Availability

Not applicable.
